# Parvalbumin Interneurons of Central Amygdala Regulate the Negative Affective States and the Expression of Corticotrophin-Releasing Hormone During Morphine Withdrawal

**DOI:** 10.1093/ijnp/pyw060

**Published:** 2016-07-06

**Authors:** Li Wang, Minjie Shen, Changyou Jiang, Lan Ma, Feifei Wang

**Affiliations:** State Key Laboratory of Medical Neurobiology, School of Basic Medical Sciences and Institutes of Brain Science, and the Collaborative Innovation Center for Brain Science, Fudan University, Shanghai, China (Drs L Wang, Ma, F Wang, and Shen, and Mr Jiang); Department of Assisted Reproduction, Shanghai Ninth People’s Hospital Affiliated Shanghai Jiao Tong University School of Medicine, Shanghai 200011, China (Dr L Wang)

**Keywords:** central amygdala, CRH, morphine withdrawal, negative affective states, PV

## Abstract

**Background:**

The central nucleus of the amygdala (CeA) is a crucial component of the neuronal circuitry mediating aversive emotion. Its role in the negative affective states during drug withdrawal includes changes in opioidergic, GABAergic, and corticotropin-releasing factor neurotransmission. However, the modulation of the neurobiological interconnectivity in the CeA and its effects in the negative reinforcement of drug dependents are poorly understood.

**Method:**

We performed electrophysiological recordings to assess the membrane excitability of parvalbumin (PV)^+^ interneurons in the CeA during chronic morphine withdrawal. We tested the morphine withdrawal–induced negative affective states, such as the aversive (assessed by conditioned place aversion), anxiety (assessed by elevated plus maze), and anhedonic-like (assessed by saccharin preference test) behaviors, as well as the mRNA level of corticotropin-releasing hormone (CRH) via optogenetic inhibition or activation of PV^+^ interneurons in the CeA.

**Result:**

Chronic morphine withdrawal increased the firing rate of CeA PV^+^ interneurons. Optogenetic inhibition of the activity of CeA PV^+^ interneurons attenuated the morphine withdrawal–induced negative affective states, such as the aversive, anxiety, and anhedonic-like behaviors, while direct activation of CeA PV^+^ interneurons could trigger those negative affective-like behaviors. Optogenetic inhibition of the CeA PV^+^ interneurons during the morphine withdrawal significantly attenuated the elevated *CRH* mRNA level in the CeA.

**Conclusion:**

The activity of PV^+^ interneurons in the CeA was up-regulated during chronic morphine withdrawal. The activation of PV^+^ interneurons during morphine withdrawal was crucial for the induction of the negative emotion and the up-regulation of *CRH* mRNA levels in the CeA.

## Introduction

Drug addiction is defined as compulsive use of drugs (Hyman et al., 2006), and relapse is the primary problem in treating drug abuse (O’Brien, 1997). Addiction to drugs such as opiates depends not only on their positive reinforcement and hedonic effects, but also on avoidance of the negative, aversive consequences of withdrawal (Solomon and Corbit, 1974; Koob et al., 1989). A severe opiate withdrawal syndrome in opiate addicts is composed of influenza-like somatic signs and negative affective symptoms such as anxiety, dysphoria, and anhedonia (American Psychiatric Association, 2000). The negative affective consequences of opiate withdrawal can enhance the incentive value of the drug and contribute to the maintenance of drug-seeking behavior (Hutcheson et al., 2001; Koob and Le Moal, 2005; Kenny et al., 2006). Thus relief of opiate withdrawal–induced negative affective states might play an important role in alleviating the relapse of opioid addiction, and elucidating the neuronal mechanism modulating the negative affective states during drug withdrawal should be an important step in solving the relapse of drug addiction.

The central nucleus of the amygdala (CeA) is a functionally interconnected region of the extended amygdala that integrates emotional, learning, motivational, nociceptive, and decision-making information (Sirohi et al., 2012), and may represent a common anatomical substrate to produce the negative emotional states that promote negative reinforcement mechanisms associated with the development of addiction (Weiskrantz, 1956; LeDoux, 2000; Phelps and LeDoux, 2005). The CeA consists of a dense network of interneurons as well as GABAergic projection neurons (McDonald, 1982). The complex interconnectivity of CeA, which plays prominent roles in fear and anxiety (Rodrigues et al., 2004; Ciocchi et al., 2010; Davis et al., 2010; Haubensak et al., 2010; Tye et al., 2011), has been discovered recently by the emerging optogenetic technique. The CeA has been indicated to play a critical role in opioid withdrawal–induced negative affective states, as well as the stress-induced relapse. A role for the CeA in the aversive effects of drug withdrawal includes changes of opioidergic, GABAergic, and corticotropin-releasing hormone (CRH) neurotransmission in the CeA. The CRH system in the CeA is activated during acute cocaine, alcohol, opioid, and nicotine withdrawal, as measured by in vivo microdialysis and neuropharmacological probes (Contarino and Papaleo, 2005; Papaleo et al., 2007).

As one of the biggest interneuron populations in the basolateral amygdaloid nucleus (BLA) (McDonald and Mascagni, 2001; Bocchio et al., 2015), the PV^+^ interneurons play an important role in controlling emotional behavior by regulating the activity of principal neurons through providing inhibitory postsynaptic potential (Wilson et al., 1994; Povysheva et al., 2006), and are thought to be involved in the control of fear by targeting the perisomatic region of principal neurons (Bienvenu et al., 2012), inhibiting and synchronizing their firing (Popescu and Pare, 2011). Although PV^+^ interneurons are not the biggest interneuron population in the CeA, their function in the CeA has been indicated to be significantly related to anxiety-like behavior (Ravenelle et al., 2014). However, whether the plasticity of PV^+^ interneurons of CeA can be modulated during drug withdrawal, and their function and neurophysiological mechanism in regulating negative affective symptoms during withdrawal, are largely unknown.

In the present study, we investigated the influence of chronic morphine exposure on the activity of PV^+^ interneurons in the CeA, and then optogenetically manipulated the activity of PV^+^ interneurons in the CeA to explore their role in withdrawal-induced negative affective behaviors such as dysphoria, depressed mood, and anhedonia. When the activity of PV^+^ interneurons in the CeA was inhibited by the optogenetic approach during the morphine-withdrawal stage, we also explored the changes of CRH in the CeA, which is not only a key mediator of the behavior responses to stressors but also a related emotional hormone (Koob, 2010).

## Materials and Methods

### Animals and Housing

*Parvalbumin(PV*)-*Cre* **(*#***008069), *CRH* ^*tm1(cre)Zjh*^ (#012704), *Gt(ROSA)26Sor* ^tm27.1(CAG-COP4 *H134R/tdTomato)Hze^ (#012567), and *Gt(ROSA)26Sor* ^*tm35.1(CAG-aop3/GFP*)^ (#012735) mice were purchased from the Jackson Laboratory. All mice were bred onto a C57BL/6J genetic background. We generated the conditional expression mice: (1) to label and excite the PV^+^ interneurons by the 473nm laser stimulation, we generated the *PV-Cre*; *Gt(ROSA)26Sor* ^tm27.1(CAG-COP4*H134R/tdTomato)Hze^ (simplified as *PV*;*ChR2 - tdTomato* ^*(+/*-)^) mice by crossing *PV*-*Cre* with *Gt(ROSA)26Sor* ^tm27.1(CAG-COP4*H134R/tdTomato)Hez^ mice; (2) to label and inhibit the PV^+^ interneurons by the 594nm laser stimulation, we generated *PV-Cre*; *Gt(ROSA)26Sor* ^*tm35.1(CAG-aop3/GFP) Hze*^ (simplified as *PV*;*Arch-GFP* ^*(+/*-)^) mice by crossing *PV*-*Cre* with *Gt(ROSA)26Sor* ^*tm35.1(CAG-aop3/GFP)Hez*^ mice; and (3) to label the CRH^+^ neurons, we generated *CRH* ^*tm1(cre)Zjh*^; *Gt(ROSA)26Sor* ^*tm35.1(CAG-aop3/GFP) Hze*^ (simplified as *CRH*;*Arch-GFP* ^*(+/*-)^) mice by crossing *CRH* ^*tm1(cre)Hez*^ with *Gt(ROSA)26Sor* ^*tm35.1(CAG-aop3/GFP)Hez*^ mice. The male offspring were used in experiments and the *PV*;*ChR2-tdTomato* ^*(-/*-)^, *PV*;*Arch-GFP* ^*(-/*-)^ littermates were used as control mice, respectively (see Figure S1A–C for a breeding scheme). Genotypes were determined by polymerase chain reaction (PCR) of mouse tail DNA samples (Zhao et al., 2011). Mice were housed in groups on a 12h light/dark cycle with food and water available *ad libitum*. All animal treatments were strictly in accordance with the National Institutes of Health Guide for the Care and Use of Laboratory Animals and Use of Laboratory Animals and were approved by the Animal Care and Use Committee of School of Basic Medical Sciences of Fudan University. The male mice aged 8–10 weeks were used for all behavioral tests.

### Stereotaxic Surgery and Laser Stimulation

Male mice aged 8–12 weeks were anesthetized with choral hydrate (40mg/kg, i.p.), placed in a stereotactic instrument, given a craniotomy, and cannula guides (Plastics One) were implanted to the brain dorsal to bilateral CeA and secured with dental cement. The intended stereotaxic coordinates were: Anteroposterior -1.1mm; Mediolateral ± 2.4mm; Dorsoventral -4.1mm (Tye et al., 2011; Li et al., 2013). All mice were given at least 14 days to recover before the behavioral experiments. To manipulate neuronal activity during behavioral experiments, two 200 μm diameter optical fibers were inserted through the cannula to deliver lasers to the CeA (DV -4.4mm). Optical fibers were attached through a ferrule connector/physical connection (FC/PC) adaptor to a 473nm blue laser or 594nm yellow laser diode (Brain-King), light pulses were generated through a Master-8 pulse stimulator (AMPI) to deliver light trains at 20 Hz and 5ms pulse-width (Zhao et al., 2011) for 473nm light, and constant light for 594nm light (Huff et al., 2013) in the experiments. The light intensity of 473nm and 594nm laser at the fiber tip was ~8–10 mW, measured using a light sensor (Thorlabs) before implantation. After the behavioral tests, the mice were sacrificed and the optical tracts and the cannula placements were confirmed by histology (Figure S2A). The behavioral data were only used from the mice in which the tracts were in the accurate position.

### Morphine Withdrawal-Induced Conditioned Place Aversion

Conditioned place aversion (CPA) is a recognized paradigm of negative affective learning. The CPA procedure induces place aversion when the mouse pairs the equipment environment with the negative effects of morphine withdrawal syndrome. The CPA apparatus consists of a two-chamber apparatus (Med-Associates) with distinct tactile environments to maximize contextual differences. One chamber of the box has a wire mesh floor while the other chamber has a grid rod floor. During the test sessions, the opened door (7×5cm) in the central partition allows the mice to enter both sides of the apparatus, whereas during the conditioning trials the individual compartments are closed off from each other. The mice were put on food and water restriction for 1 hour before each session. On day 1, each mouse was allowed to explore freely in the entire CPA apparatus for 20min and time spent in each of the two compartments was measured (pre-test). Within each genotype, the mice were divided into two groups. One group was assigned to receive saline and the other to receive increasing morphine doses progressively (20–80mg/kg). Every 12h (10:00 and 22:00 hours) the mice were treated with saline or morphine according to the following protocol: day 2, 10mg/kg; day 3, 20mg/kg; day 4, 40mg/kg; day 5, 60mg/kg; day 6, 80mg/kg; and day 7, 80mg/kg (only one injection in the morning). CPA conditioning trials took place on days 4–7, when the morphine-treated mice were in an opiate withdrawal state. For this purpose, 10h after the evening injection, while the drug was almost cleared in the body and induced the maximal expression of somatic opiate withdrawal signs, the mice were confined for 30min into their preferred compartment of the CPA apparatus, determined during the preconditioning test. Post-conditioning tests of 20min with ~8–10 mW of constant 594nm light took place 6 days after the last conditioning trial (day 13), when somatic opiate withdrawal signs had largely dissipated in both genotypes (Papaleo et al., 2008; Ingallinesi et al., 2012).

### Optogenetic Activation-Induced Conditioned Place Aversion

Conditioned place aversion, induced by laser stimulation of CeA PV^+^ interneurons, was also assessed using the two-chamber apparatus (Med-Associates). A manual guillotine door separates the two chambers. On day 1, mice were placed in one of the chambers and allowed to freely explore the entire apparatus for 20min (pre-test). On days 2–4, mice were given a 473nm laser stimulation (20Hz frequency, 5ms duration, delivered at 5min duration with 5min intervals) through the embedded cannula in the CeA in the morning when confined to one chamber for 20min. In the afternoon they were given an equivalent false optical fiber without a laser, then confined to the other chamber for 20min. On day 5, mice were allowed to freely explore the entire apparatus for 20min (test). The time spent in each chamber was recorded during the pre-test and test sections. The CPA score was defined as the time (in seconds) spent in the 473nm laser-paired chamber minus the time spent in the no laser–paired chamber.

### Elevated Plus Maze

Six days after the last morphine injection (day 13), when somatic opiate withdrawal signs had largely dissipated in both genotypes, we first use the elevated plus maze (EPM) to test the morphine withdrawal–induced anxiety-like behavior. The mice were put on food and water restrictions for 1 hour before the test. The elevated plus maze consisted of four arms (34.5cm length × 6.3cm width × 19.5cm height) elevated 75cm above the floor. Two of the arms had 20cm high dark walls (closed arms) and two had 0.8cm high ledges (open arms). The arms were angled at 90° to each other. The apparatus was placed in a quiet and dimmed room. The mice were placed in the center of the maze facing the open arm and were allowed to explore the equipment freely for 5min. The apparatus was wiped with water and dried between tests to ensure the absence of olfactory cues. The EthoVision XT 8.5 video tracking program was used to track mouse location, velocity, and movement of head, body, and tail. Laser stimulation protocols are specified by groups. Bilateral illumination of PV^+^ interneurons in the CeA was delivered at 5min duration with constant 594nm light of 8–10 mW at the tip of the fiber.

Another group of mice without morphine injection and withdrawal were used to test the function of CeA PV^+^ interneurons on anxiety when directly given a bilateral 473nm laser with 20Hz frequency, 5ms duration delivered at 3min duration after 3min laser off.

### Saccharin Preference Test

A morphine withdrawal–induced decrease in the preference for saccharin is thought to reflect anhedonia-like states. This experiment had training and testing sessions. During training, mice were restricted from food and water for 12h and trained to consume 1% (w/v) saccharin solution before the first morphine injection. Saccharin preference was tested 1 day after the EPM or CPA test (7 days after the last morphine injection, day 14). Mice were again deprived of food and water for 12h before the 30min testing session; mice could select between two pre-weighed bottles, one with 1% (w/v) saccharin solution and the other with tap water. The saccharin preference was calculated as the following formula: saccharin preference = [saccharin solution intake (g) × 100] / [saccharin solution intake (g) + water intake (g)]. Bilateral constant light for the 594nm laser was delivered during the 30min testing session.

Another group of mice without morphine injection and withdrawal were used to test the function of CeA PV^+^ interneurons in the anhedonia when directly given bilateral 473nm laser (with 20Hz frequency, 5ms duration delivered at 5min duration with 5min interval) during the 30-minute saccharin preference test.

### Immunohistochemistry

Mice were anesthetized with choral hydrate and perfused with saline followed by 4% paraformaldehyde in 0.1M phosphate-buffered saline (PBS). The brains were removed, fixed in 4% paraformaldehyde overnight, and subjected to dehydration in increasing saccharin solutions (20–30%) at 4^º^C. The frozen coronal slices of 20 μm thicknesses were prepared and stored at -20^º^C in 20% ethanediol PBS solution containing 20% saccharin. Brain sections were incubated in 3% normal goat serum and 0.2% Triton-X for 1h. Then they were incubated in rabbit anti-Parvalbumin (Swant, Bellinzona) or mouse anti–corticotropin-releasing factor (CRF; Abcam) antibody overnight at 4ºC. Slices were rinsed in PBS then incubated in donkey anti-rabbit Cy3, Alex488, DyLight 647, or donkey anti-mouse Cy3 (Jackson Immunoresearch) for 1h and 4',6-diamidino-2-phenylindole (DAPI) for 10min, then mounted after rinsing. Images were acquired on a microscope using a 20× air objective, a 40× objective (IX-83; Olympus) or a 63× oil immersion objective (Zeiss 510; Carl Zeiss Jena).

### Electrophysiology

6 days after the last morphine injection (day 13), coronal sections (300 μm) containing CeA were cut from *PV*;*ChR2 -tdTomato* ^*(+/*-)^ mice and slices were prepared as previously described (Zhao et al., 2011). Briefly, the mice were deeply anesthetized by intra-peritoneal injection of chloral hydrate (400mg/kg, i.p.) and then transcardially perfused with cold protective artificial cerebrospinal fluid (ACSF) (92mM N-methyl-D-glucamine [NMDG], 2.5mM KCl, 1.25mM NaH_2_PO_4_, 30mM NaHCO_3_, 20mM HEPES, 25mM D-glucose, 2mM thiourea, 5mM Na-ascorbate, 3mM Na-pyruvate, 0.5mM CaCl_2_, and 10mM MgCl_2_) and then initially recovered at 32–34 ^º^C for 10–15min. The slices were transferred into a holding chamber containing room temperature carbogenated ACSF (119mM NaCl, 2.5mM KCl, 1.25mM NaH_2_PO_4_, 26mM NaHCO_3_, 12.5mM glucose, 2mM CaCl_2_, 2mM MgCl_2_, 2mM thiourea, 5mM Na-ascorbate, and 3mM Na-pyruvate), and slices were stored for 0.5–3 hours prior to being transferred to the recording chamber for use. All solutions were saturated with 95% O_2_, 5% CO_2_, and the slices were used within 6 hours after preparation.

Whole-cell current-clamp recordings were performed in ACSF at room temperature from PV-positive neurons in CeA with an EPC-10 amplifier and Pulse v8.78 software (HEKA Elektronik). Intracellular solution composition was: 126mM K-gluconate, 4mM KCl, 10mM HEPES, 4mM ATP-Mg, 0.3mM GTP-Na_2_, 10mM creatine phosphate (pH 7.2, 290–300 mOsm). At least 5min after achieving whole cell configuration, a current-step protocol (from -200 to + 200 pA, with a 10 pA increment) was run and repeated. The after-hyperpolarization potential was sampled following the first single-action potential spike. Data were filtered at 300 Hz and were analyzed by Mini Analysis Program (Synaptosoft Inc.). Spontaneous miniature excitatory postsynaptic current (mEPSC) events were recorded in the presence of ACSF containing 2 μM TTX, 50 μM bicuculline (Tocris Bioscience) at a holding potential of -70 mV. Data were filtered at 300 Hz and were analyzed by Mini Analysis Program (Synaptosoft Inc.). All electrophysiological recordings were performed and analyzed blind to genotype.

### RNA Extraction and Real-Time PCR Analysis

Mice which were given 30min 594nm laser stimulation on day 14 after the EPM or CPA test (7 days after the last morphine injection) were sacrificed and the brains were removed immediately. The CeA was dissected within 5min at 0 ^º^C by using Microm HM 650V (Thermo Fisher Scientific Inc.) according to stereotaxis coordinates from Bregma −0.9mm to −1.7mm, rinsed in PBS, frozen in TRIzol Reagent (Thermo Fisher Scientific Inc.) and stored at –70 ^º^C until extraction. Total RNA was extracted from tissues using the TRIzol Reagent according to the manufacturer’s instructions, with the inclusion of a DNase digestion step. The Superscript First-Strand Synthesis System for reverse transcription (TAKARA) was used with random primers. Quantitative real time (RT)-PCR amplification of the cDNA was performed on samples in triplicate with Power SYBR Green PCR Master Mix (TAKARA) using the Eppendorf Mastercycler ep gradient S PCR System (Eppendorf). *CRH* mRNA expression was normalized to the internal control *GAPDH*.

### Statistical Analysis

Data are expressed as means ± standard error of the mean, and analyzed by GraphPad Prism (GraphPad Software, Inc.). A Mann-Whitney U Test was used for the AP initiation, the frequency and amplitude of mEPSCs, the travel distance, velocity, saccharin preference, and EPM tests of the different genotype or treatment. Two-way reverse measures analyses of variance (ANOVA) were used to assess the spike numbers within each genotype and the effects of laser stimulation or genotype on CPA scores within each genotype and treatment (saline or morphine). Two-way ANOVA was used to examine the *CRH* mRNA level within each genotype. Bonferroni’s post hoc test was used.

## Results

### CeA PV^+^ Interneurons are Activated During the Withdrawal of Chronic Morphine Exposure

**Figure 1.**
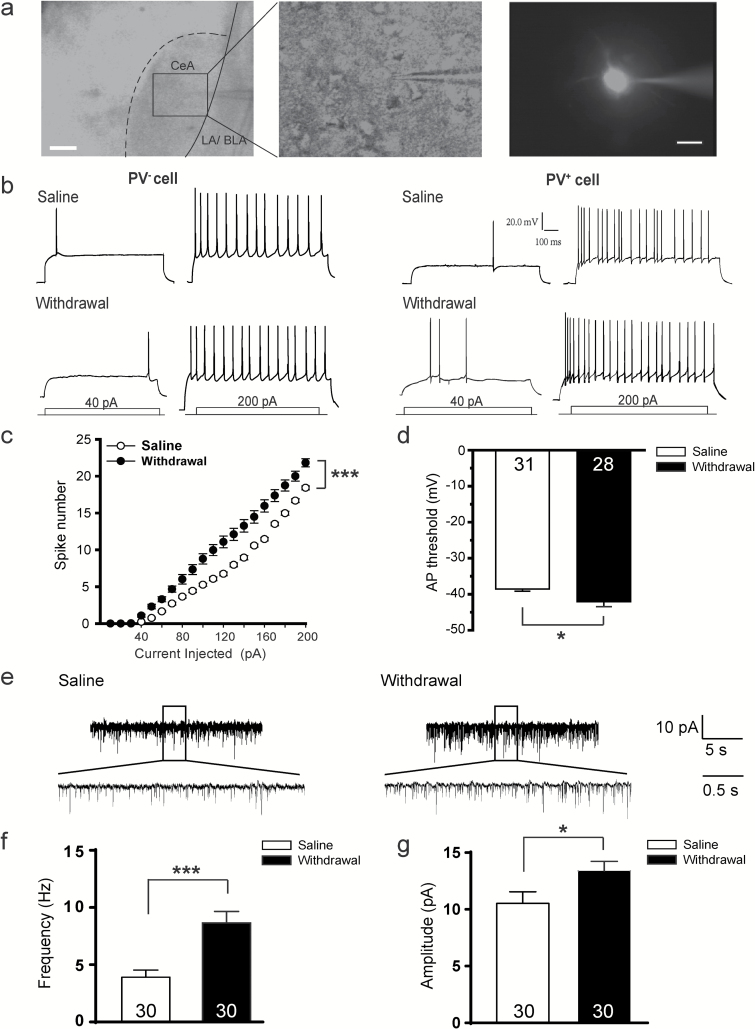
Morphine withdrawal increases the membrane excitability of parvalbumin (PV)^+^ interneurons in the central nucleus of the amygdala (CeA). (A) Left: representative images of a PV^*+*^ neuron that has been patched. Middle: the black zoom in the left was enlarged. Right: the PV^*+*^ neuron filled with Alexa Fluor-568 dye through the patch pipette after recording. Scale bar, 100 µm (left) and 20 µm (right). (B) Example of AP (action potential) trains upon current injections from PV^-^ neurons and PV^+^ interneurons in the CeA of *PV;ChR2-tdTomato* ^*(+/*-)^ transgenic mice on the 6th day from the last saline or morphine exposure. (C) Spike number of PV^+^ interneurons in the CeA after morphine withdrawal. Two-way repeated measures analyses of variance, **p < 0.001. (D) PV^+^ interneurons in the CeA exhibited significant decrease in AP threshold after withdrawal from morphine exposure. (E) Representative traces of spontaneous miniature excitatory postsynaptic currents recorded from PV^+^ interneurons in the CeA 6 days after morphine withdrawal (right) or saline control (left). (F–G) Average frequency and amplitude recorded from PV^+^ interneurons in the CeA after morphine withdrawal or saline control. Mann-Whitney U Test, *p < 0.05, **p < 0.001. Data are presented as mean ± standard error of the mean.

The membrane excitability during chronic morphine withdrawal was examined first. We recorded evoked action potential (AP) of the PV^+^ interneurons of CeA (Figure 1A) in *PV*;*ChR2-tdTomato* ^*(+/*-)^ transgenic mice that had consecutively withdrawn from the chronic morphine injection for 6 days (Ingallinesi et al., 2012). Compared with the saline controls, the membrane excitability was increased in PV^+^, but not PV^-^ interneurons (Figure 1B). The number of spikes recorded from PV^+^ interneurons in the CeA was increased relative to that from the saline control [Figure 1C, treatment × current: F (1, 56) = 17.742, *p* < 0.001]. PV^+^ interneurons exhibited a lower threshold for AP initiation (Figure 1D, *p* = 0.025), suggesting that the membrane excitability of PV^+^ interneurons was increased during chronic morphine withdrawal. No significant changes were detected in the after-hyperpolarization, spike amplitude, and half-width of PV^+^ interneurons (Figure S1D–F: D, *p* = 0.360; E, *p* = 0.289; F, *p* = 0.429), suggesting that morphine withdrawal enhanced membrane excitability without changing membrane properties of fast-spiking PV^+^ GABAergic interneurons (Hu et al., 2014).

To assess the effect of chronic morphine withdrawal on the synaptic transmission of PV^+^ interneurons of CeA, pharmacologically isolated spontaneous miniature excitatory postsynaptic currents (mEPSCs) of PV^+^ interneurons were recorded and analyzed (Figure 1E–G). We found that the frequency (Figure 1F) and amplitude (Figure 1G) of mEPSCs recorded from PV^+^ interneurons of the morphine withdrawal mice significantly increased relative to that from the saline control, indicating an up-regulation of excitatory synaptic transmission of PV^+^ interneurons of CeA during the chronic morphine withdrawal phase. These results suggested the PV^+^ interneurons in the CeA were activated during the chronic morphine withdrawal phase, accompanied with the negative affective states.

### Optogenetic Inhibition of PV^+^ Interneurons Activity in the CeA During the Morphine Withdrawal Attenuates Negative Affective States

**Figure 2.**
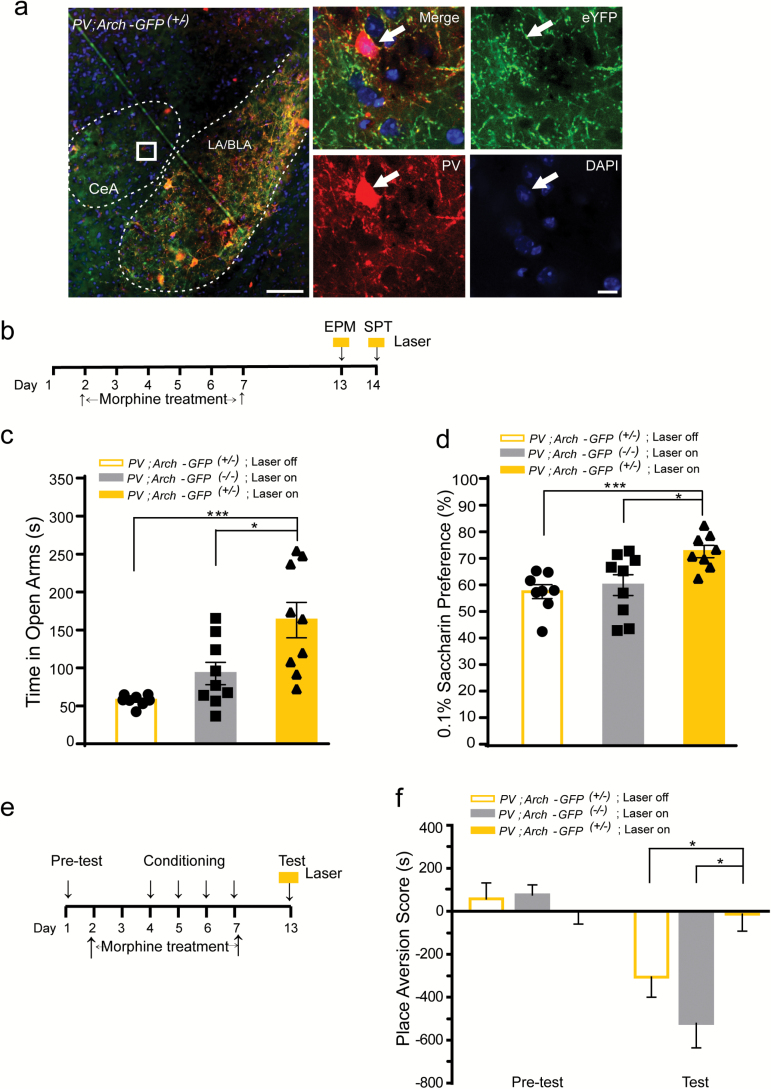
Optogenetic inhibition of the parvalbumin (PV)^+^ interneurons in the central nucleus of the amygdala (CeA) during the morphine withdrawal attenuates its induced negative affective states. (A) Representative image of the amygdala of mice stained with the PV antibody. White dashes indicate the CeA and lateral amygdaloid nucleus/basolateral amygdaloid nucleus, anterior part (LA/BLA) structure. The white box was enlarged on the right. Arrows indicate the containment of PV antibody with the Arch-GFP^+^ neurons. Green, green fluorescent protein (GFP); red, PV; blue, 4',6-diamidino-2-phenylindole (DAPI). Scale bar: 200 µm (left), 20 µm (right). (B–F) *PV*;*Arch-GFP* ^*(+/*-)^ and *PV*;*Arch-GFP* ^*(-/*-)^ mice were administered increasing morphine dose for 7 days and subjected to withdrawal for 6–7 days. (B) The schematic experimental schedule of the EPM and SPT tests. (C) Time spent in the open arms of the 5-min EPM test in the *PV;Arch-GFP* ^*(+/*-)^ mice with or without the 594nm laser stimulation, and *PV;Arch-GFP* ^*(-/*-)^ with the 594nm laser stimulation. *p < 0.05, **p < 0.001, Mann-Whitney U Test. (D) Saccharin preference test in the *PV;Arch-GFP* ^*(+/*-)^ mice with or without the 594nm laser stimulation, and in the *PV;Arch-GFP* ^*(-/*-)^ mice with the 594nm laser stimulation. *p < 0.05, **p < 0.001, Mann-Whitney U Test. (E) The schematic experimental schedule of the CPA test. (f) Place aversion scores in the *PV;Arch-GFP* ^*(+/*-)^ mice with or without the 594nm laser stimulation, and in the *PV;Arch-GFP* ^*(-/*-)^ mice with the 594nm laser stimulation. *p < 0.05, two-way repeated measures analyses of variance with Bonferroni’s post hoc test. Data are presented as mean ± standard error of the mean.

We inhibited the activity of CeA PV^+^ interneurons, of which Arch-GFP fusion protein is selectively expressed in PV^+^ interneurons, by 594nm laser stimulation in the *PV*;*Arch-GFP* ^*(+/*-)^ mice. The expression of Arch-GFP in the CeA is shown by immunofluorescence images in Figure 2A. As compared with lateral amygdaloid nucleus/basolateral amygdaloid nucleus, anterior part (LA/BLA), the CeA does not have as much PV^+^ soma indicated by the PV antibody staining, but has a large amount of dendritic protrusions of PV^+^ interneurons (Figure 2A) indicated by the Arch-eGFP expression. The localization of Arch-eGFP in PV^+^ cells in the CeA confirmed that the CeA has a moderate level of PV^+^ interneurons.

Opiate withdrawal–induced affective-like states are composed of dysphoria, anxiety, and anhedonia. An intermittent and progressively-increasing-dose morphine injection procedure was given to the mice to mimic the drug intake patterns of opiate addicts. Previous studies showed that 6 days after the last morphine injection, mice suffered from the morphine withdrawal–induced negative affective states such as aversive, increased anxiety, and anhedonia (Papaleo et al., 2007; Ingallinesi et al., 2012). To examine the possible role of the CeA PV^+^ interneurons in the morphine withdrawal–induced negative affective states, CPA, EPM, and SPT experiments were carried out, and the activity of PV^+^ interneurons in the CeA of *PV*;*Arch-GFP* ^*(+/*-)^ mice was inhibited by the bilateral 594nm laser stimulation during these tests.

We used the EPM to assess anxiety-like behavior after 6 days of withdrawal from morphine (Zanos et al., 2014). We found that the *PV*;*Arch-GFP* ^*(+/*-)^ mice with 594nm laser stimulation (laser on) spent increased time in the open arm of the EPM, when compared with the *PV*;*Arch-GFP* ^*(+/*-)^ mice without laser stimulation (laser off) or the laser-stimulated *PV*;*Arch-GFP* ^*(-/*-)^ group (Figure 2C), indicating that inhibiting the activity of CeA PV^+^ interneurons could attenuate morphine withdrawal–increased anxiety levels.

The SPT was used to create depressive-like behavior to investigate the anhedonic state of morphine withdrawal (Casarotto and Andreatini, 2007; Pisu et al., 2011; Fadaei et al., 2015). After the 7-day morphine withdrawal, the *PV*;*Arch-GFP* ^*(+/*-)^ mice showed increased saccharin preference during the 594nm laser stimulation, as compared with the *PV*;*Arch-GFP* ^*(+/*-)^ mice without laser stimulation and the laser-stimulated *PV*;*Arch-GFP* ^*(-/*-)^ mice (Figure 2D). These results suggest that inhibiting the activity of CeA PV^+^ interneurons could attenuate the morphine withdrawal–induced anhedonic-like behavior.

CPA is a typical paradigm of negative affective states associated with opiate withdrawal (Contarino and Papaleo, 2005). In the pre-test section of the CPA experiment, both the *PV*;*Arch-GFP* ^*(+/*-)^ mice and the control littermates spent similar time in the two compartments of the CPA apparatus. After four sessions of morphine withdrawal conditioning, mice were given the 594nm laser stimulation in the CeA during the test session (Figure 2E). We found that both *PV*;*Arch-GFP* ^*(-/*-)^ mice given laser stimulation and *PV*;*Arch-GFP* ^*(+/*-)^ mice without laser stimulation avoided and spent less time in the apparatus previously paired with the morphine withdrawal; however, the laser-stimulated *PV*;*Arch-GFP* ^*(+/*-)^ mice did not show any aversion to the environmental cues paired with morphine withdrawal (Figure 2F; laser × treatment interaction effect, F_1, 12_ = 4.60, *p* < 0.05; genotype × treatment interaction effect, F_1, 10_ = 11.37, *p* < 0.05). Optogenetic inhibition of the activity of PV^+^ interneurons in the CeA alone did not have any effect on travelling velocity (Figure S2B) and distance travelled (Figure S2C).

These results suggest that inhibiting the activity of PV^+^ interneurons in the CeA abolished the expression of aversion, as well as alleviated morphine withdrawal–induced anxiety and anhedonic-like behaviors, when exposure in the environmental cues was paired with the dysphoria state induced by morphine withdrawal.

### Optogenetic Activation of CeA PV^+^ Interneurons Can Induce Similar Negative Affective States as Morphine Withdrawal

**Figure 3.**
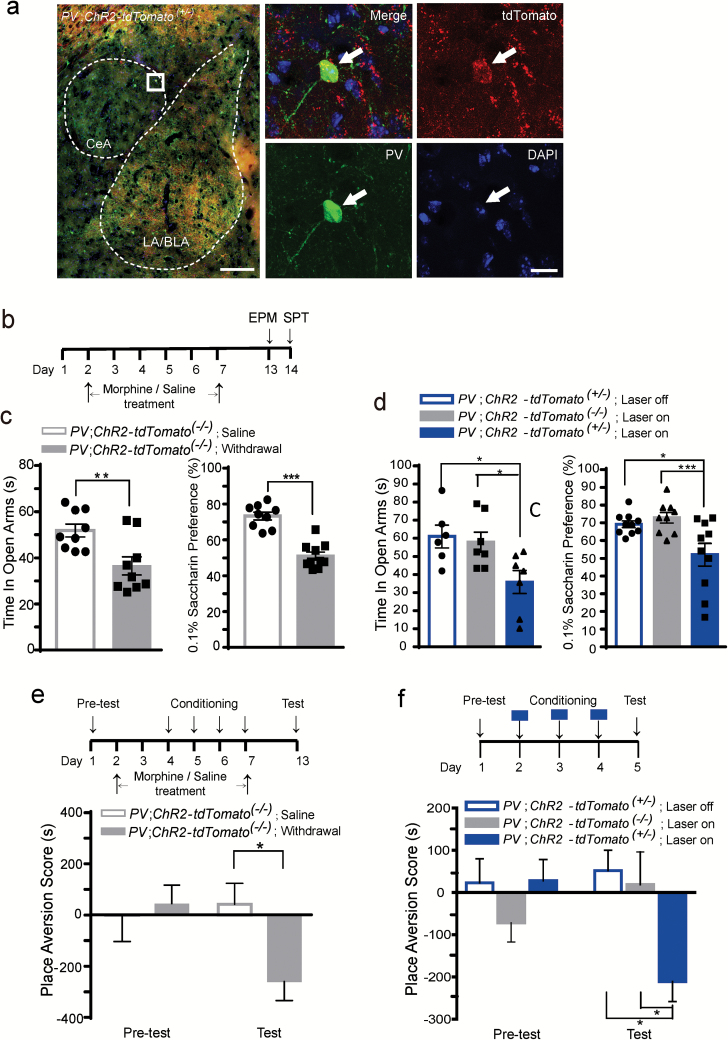
Optogenetic activation of the parvalbumin (PV)^+^ interneurons in the central nucleus of the amygdala (CeA) mimics negative affective states during morphine withdrawal. (A) Representative image of the amygdala of *PV*;*ChR2-tdTomato* ^*(+/*-)^ mice stained with the PV antibody. White dashes indicate the CeA and LA/BLA structure. The white box was enlarged on the right. Arrows indicate the co-localized PV antibody with the ChR2-tdTomato^+^ neurons. Red, tdTomato; green, PV; blue, DAPI. Scale bar: 200 µm (left), 20 µm (right). (B–D) The elevated plus maze (EPM) and saccharine preference (SPT) tests. (B) The schematic experimental schedule of the EPM and SPT tests for the morphine withdrawal group. (C) The time spent in the open arms of the EPM (left) or the SPT (right) in the *PV*;*ChR2-tdTomato* ^*(-/*-)^ mice in the morphine withdrawal and saline groups. **p < 0.01, ***p < 0.001, Mann-Whitney U Test. (D) The time spent in the open arms of the EPM (left) or the SPT (right) in the *PV*;*ChR2-tdTomato* ^*(+/*-)^ mice with or without the 473nm laser stimulation, and in the *PV*;*ChR2-tdTomato* ^*(+/*-)^ mice with the 473nm laser stimulation. *p < 0.05, ***p < 0.001, Mann-Whitney U Test. (E) The conditioned place aversion (CPA) test of the *PV*;*ChR2-tdTomato* ^*(-/*-)^ mice in the morphine withdrawal or saline groups. (F) The CPA test of the *PV*;*ChR2-tdTomato* ^*(+/*-)^ mice with or without the 473nm laser stimulation, and of the *PV*;*ChR2-tdTomato* ^*(+/*-)^ mice with the 473nm laser stimulation during the conditioning sessions. Upper panel is the schematic experimental schedule. *p < 0.05, two-way repeated measures analyses of variance with Bonferroni’s post hoc test. Data are presented as mean ± standard error of the mean.

Since inhibiting the activity of CeA PV^+^ interneurons during morphine withdrawal could alleviate the morphine withdrawal–induced affective states, we speculated that direct activation of the CeA PV^+^ interneurons might trigger similar negative emotions as the morphine withdrawal. Therefore we activated the CeA PV^+^ interneurons by 473nm laser stimulation in *PV*;*ChR2-tdTomato* ^*(+/*-)^ mice, in which the ChR2(H134R)-tdTomato fusion protein was selectively expressed in the PV^+^ interneurons (Figure 3A), and found the 473nm laser stimulation in the CeA of *PV*;*ChR2-tdTomato* ^*(+/*-)^ mice triggered dramatically increased c-fos^+^ cells in the CeA, but not the LA/BLA (Figure S3A–B).

Our results (Figure 3C) show that in *PV;ChR2-tdTomato* ^*(-/*-)^ mice as compared with the saline group, morphine withdrawal could reduce the time spent on the open arm in the EPM (Figure 3C, left panel) and reduce saccharin preference (Figure 3C, right panel), indicating morphine withdrawal could increase anxiety levels and anhedonia-like behavior. To assess the extent to which artificial optogenetic activation of PV^+^ interneurons in the CeA can mimic the effect of morphine withdrawal, we directly activated the CeA PV^+^ interneurons during the EPM and SPT tests. The results showed that 473nm laser stimulation of CeA in *PV;ChR2-tdTomato* ^(+/-)^ mice decreased their time spent on the open arm and their saccharin preference, as compared with the *PV;ChR2-tdTomato* ^*(+/*-)^ mice without laser stimulation or with the laser-stimulated *PV;ChR2-tdTomato* ^*(-/*-)^ mice (Figure 3D). Activation of PV^+^ interneurons in CeA did not have any effect on the velocity (Figure S2D, *p* = 0.31) and distance travelled (Figure S2E, *p* = 0.13) of the mice.

The morphine withdrawal–induced CPA test showed that after four sessions of morphine withdrawal conditioning the *PV;ChR2-tdTomato* ^*(-/*-)^ *mice* avoided and spent less time in the apparatus previously paired with the morphine withdrawal in the test session (Figure 3E, genotype × treatment interaction effect: F_1, 14_ = 6.96, *p* < 0.05). To assess to whether optogenetic activation of PV^+^ interneurons in the CeA can induce CPA as morphine withdrawal, we directly activated PV^+^ interneurons in the CeA during the conditioning. During the 20-min pretest session (day 1), time spent in the two compartments of the CPA apparatus was similar between *PV*;*ChR2-tdTomato* ^*(+/*-)^ or *PV*;*ChR2-tdTomato* ^*(-/*-)^ mice. During the conditioning sessions (days 2–4), one chamber of the apparatus was paired with or without the 473nm laser stimulation for 20min. On the test day (day 5), the time spent exploring the laser-paired chamber of the *PV*;*ChR2-tdTomato* ^*(+/*-)^ mice was significantly decreased, but the *PV*;*ChR2-tdTomato* ^*(+/*-)^ mice without laser stimulation (Figure 3F; laser × treatment interaction effect, F_1, 16_ = 6.06, *p* < 0.05) and the laser-stimulated *PV*;*ChR2-tdTomato* ^*(-/*-)^ mice (Figure 3F; genotype × treatment interaction effect, F_1, 12_ = 13.12, *p* < 0.05) did not develop CPA, indicating the *PV*;*ChR2-tdTomato* ^*(+/*-)^ mice developed an aversion for the laser-paired chamber. These results indicate the optogenetic-activating PV^+^ interneurons in the CeA could trigger similar negative affective states as morphine withdrawal, such as increased anxiety levels and anhedonic-like and aversive behaviors.

### Optogenetic Inhibition of CeA PV^+^ Interneurons During Morphine Withdrawal Down-Regulates the *CRH* mRNA Level in the CeA

The CRF (also known as corticotropin-releasing hormone, CRH) system coordinates not only neuroendocrine and autonomic responses to stressors (Rivier et al., 1982; Koob, 1999), but also to substance dependence (Rouibi and Contarino, 2013). The CRH receptor antagonists can ameliorate the negative affective-like states associated with alcohol, cocaine, or opiate withdrawal (Sarnyai et al., 1995; Valdez et al., 2002; Contarino and Papaleo, 2005; Stinus et al., 2005; Papaleo et al., 2008). Up-regulation of the CRH brain stress system in the extended amygdala has been observed in rodents, nonhuman primates, and humans during abstinence from drugs of abuse, including tobacco (Sarnyai et al., 2001; Koob and Volkow, 2010). The nicotine withdrawal–induced increase of CRH release in the CeA leads to negative affective states that contribute to increased nicotine intake (George et al., 2007; Bruijnzeel et al., 2009, 2012; Qi et al., 2014; Cohen et al., 2015).

**Figure 4.**
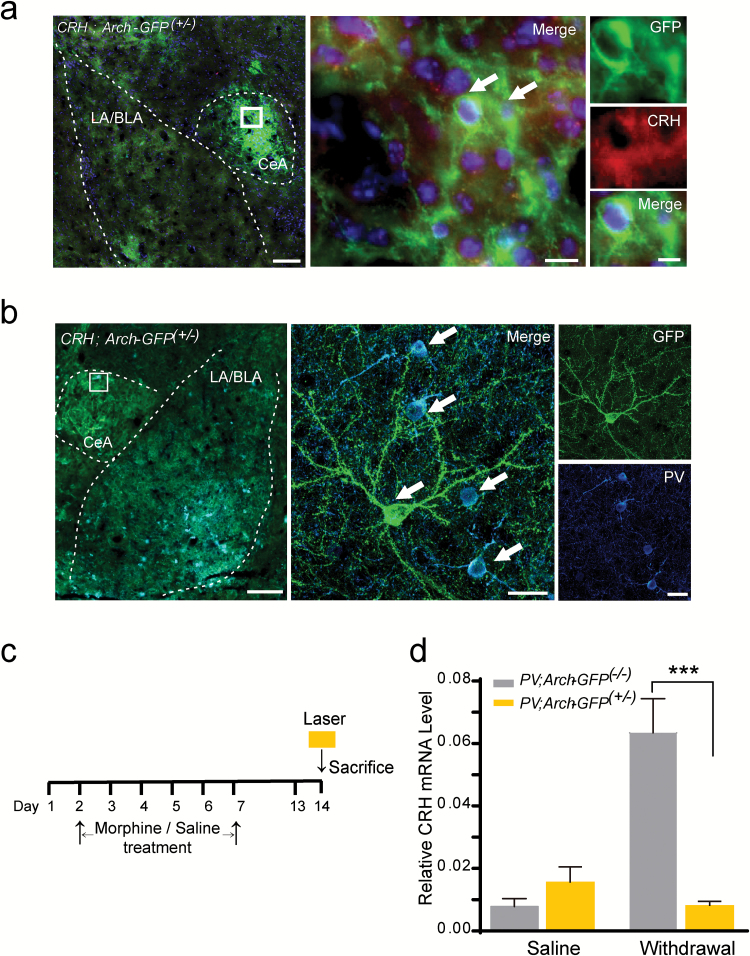
Corticotropin-releasing hormone (CRH)^+^ neurons localize in the central nucleus of the amygdala (CeA) and the *CRH* mRNA level is regulated by the activity of parvalbumin (PV)^+^ interneurons. (A) Representative image of the amygdala of *CRH*; *Arch-GFP* ^*(+/*-)^ mice stained with the CRH antibody. White dashes indicate the CeA and LA/BLA structure. The white box was enlarged on the right. Arrows indicate the containment of the CRH antibody with the Arch-GFP^+^ neurons. Green, GFP; red, CRH; blue, DAPI. Scale bar: 100 µm (left), 10 µm (middle), 5 µm (right). (B) Representative image of the amygdala of *CRH*; *Arch-GFP* ^*(+/*-)^ mice stained with the PV antibody. The white dashes indicate the CeA and LA/BLA structure. The white box was enlarged on the right. Arrows indicate the colocalization of PV antibody with the Arch-GFP fluorescent. Green, GFP; cyan, PV; blue, DAPI. Scale bar: 200 µm (left), 20 µm (middle), 20 µm (right). (C) The schematic experimental schedule. The CeA was dissected from *PV;Arch-GFP* ^*(+/*-)^ and control mice after the 30-min 594nm laser stimulation on the 7th day after the last saline or morphine exposure. (D) Quantification of the expression of the *CRH* mRNA level in the CeA of the *PV*;*Arch-GFP* ^*(+/*-)^ and *PV*;*Arch-GFP* ^*(-/*-)^ mice, both with the 30-min laser stimulation on the 7th day after saline and morphine exposure. ***p < 0.001, two-way analyses of variance with Bonferroni’s post hoc test. Data are presented as mean ± standard error of the mean.

Here, we generated *CRH*;*Arch-GFP* ^*(+/*-)^ mice that expressed the Arch-GFP fusion protein specifically directed by the mouse *CRH* promoter/enhancer (Figure 4A). We found that the CeA had a dense Arch-GFP fluorescence signal (Figure 4A, left), and this Arch-GFP fusion protein localized with CRH^+^ cells as indicated by CRH antibody staining (Figure 4A, middle and right), indicating there is a large amount of CRH^+^ neurons in the CeA. Furthermore, we found that the PV^+^ and CRH^+^ neurons had a close connection in the CeA (Figure 4B), and the cell body of PV^+^ interneurons were circled or crossed by the dendrites of CRH^+^ neurons (Figure 4B, middle and right).

To assess whether the modulation of morphine withdrawal–induced negative affective states by the CeA PV^+^ interneurons might involve the CRH system of the CeA, we carried out a real-time PCR assay to measure the effect of optogenetic inhibition in the activity of CeA PV^+^ interneurons on the *CRH* mRNA level of the CeA during the morphine withdrawal (Figure 4C). We found there was no significant difference of the *CRH* mRNA level between the laser stimulated *PV;Arch-GFP* ^*(+/*-)^ and the *PV*;*Arch-GFP* ^*(-/*-)^ control mice in the saline treatment groups. However, in the chronic morphine–treated groups, the *CRH* mRNA of the control mice was markedly increased as compared with the saline group, while optogenetic inhibition of the activity of CeA PV^+^ interneurons in the *PV*;*Arch-GFP* ^*(+/*-)^ mice significantly decreased the *CRH* mRNA level in the CeA (Figure 4D; genotype × treatment interaction effect, F(1, 20) = 44.95, *p* < 0.001) as compared with the control mice, suggesting that the activation of CeA PV^+^ interneurons may be involved in the regulation of the expression level of corticotropin-releasing hormones during morphine withdrawal.

## Discussion

In this study, we used an optogenetic approach to examine the role of CeA PV^+^ interneurons in the regulation of the morphine withdrawal–induced negative affective states. We found that optogenetic inhibition of the CeA PV^+^ interneurons’ activity during morphine withdrawal was sufficient to impair morphine withdrawal–induced aversion, anxiety, and anhedonic-like behavior. Instead, direct optogenetic activation of the CeA PV^+^ interneurons of *PV*;*ChR2-tdTomato* ^*(+/*-)^ mice could mimic several morphine withdrawal–like affective states. These results suggest that the CeA PV^+^ interneurons positively regulate morphine withdrawal–induced negative affective states. Optogenetic inhibition of CeA PV^+^ interneurons activity within morphine withdrawal could down-regulate the *CRH* mRNA level, which was up-regulated and correlated with the negative affective-like states in the CeA during opiate withdrawal. Therefore, the activation of CeA PV^+^ interneurons might mediate the morphine withdrawal–induced negative affective states, and is related with up-regulation of the CRH system in the CeA.

Although the CeA has a few soma of PV^+^ interneurons, PV^+^ interneurons might extend a large amount of dendrites or/and axons, which may form extensive synaptic connections with other neurons in the CeA. It has been reported that few PV^+^ interneurons are shown in the CeA by immunoreactive methods in rats (Zahm et al., 2003; Ravenelle et al., 2014). The immunohistochemical images of Zahm et al. (2003) show a small quantity cell body of PV^+^ interneurons in the CeA, which is consistent with our immunofluorescence results using PV antibody. Fluorescent fusion protein Arch-eYFP and ChR2-tdTomato expressed on the cell membrane and could label the dendritic structure clearly, and we saw large amount of dendrites or/and axons of PV^+^ interneurons in the CeA of *PV*;*Arch-GFP* ^*(+/*-)^ and *PV*;*ChR2-tdTomato* ^*(+/*-)^ reporter mice. These data hint that the limited PV^+^ interneurons of the CeA might have extensive connections with other neurons in the CeA and regulate the activity of other neurons through providing inhibitory postsynaptic potential. It has been reported that PV^+^ interneurons of the striatum (Cowan et al., 1990; Kawaguchi, 1993) could affect overall input-output functions in a manner disproportionate to their relatively small numbers (Zahm et al., 2003). The anatomical and electrophysiological evidence has shown that PV^+^ interneurons are electronically coupled (Kita et al., 1990; Koos and Tepper, 1999) and activation of a single PV^+^ interneuron may exert this robust inhibitory influence on up to 400 projection neurons (Koos and Tepper, 1999). Therefore it is no wonder that the limited PV^+^ interneurons in the CeA might also have strong effects on the morphine withdrawal–induced negative affective-like states.

The relationship between CRH and the negative affective-like states associated with alcohol, cocaine, or opiate withdrawal has been widely reported. CRH^+^ neurons in the CeA send dense projections to the locus coeruleus (LC), and stimulation of CRH^+^ terminals in the LC induced aversive and anxiogenic behaviors (McCall et al., 2015). Here we found that a large amount of CRH^+^ interneurons existed in the CeA and had a close connection with the PV^+^ interneurons, so we explored the change of *CRH* mRNA levels in the CeA via optogenetic inhibition of the CeA PV^+^ interneurons during morphine withdrawal. Optogenetic inhibition of CeA PV^+^ interneurons down-regulated the *CRH* mRNA level and morphine withdrawal–induced negative affective states. Inhibition of the activity of PV^+^ interneurons in the CeA has no effect on the *CRH* mRNA level in the saline group, indicating the activity of PV^+^ interneurons might be low in the physiological states and is dysregulated in the morphine withdrawal state.

Besides the PV^+^ and CRH^+^ neurons in the CeA, there are also a large amount of somatostatin-positive interneurons (Li et al., 2013), PKC-δ^+^ neurons (Haubensak et al., 2010), and GABAergic medium spiny neurons. It is quite possible that PV^+^ interneurons influence the activity of CRH^+^ neurons indirectly. The PKC-δ^+^ neurons, which are a subpopulation of GABA-containing neurons and are located in the lateral subdivision of the central amygdala, gate CEm output to control the level of conditioned freezing and anxiety (Haubensak et al., 2010). Whether the PV^+^ interneurons in CeA regulate the morphine withdrawal–induced negative affective states through PKC-δ^+^ neurons will be assessed in future.

Therefore, our study discovered that the CeA PV^+^ interneurons were activated during the withdrawal of chronic morphine exposure, and optogenetic inhibition of the PV^+^ interneurons in the CeA attenuated the morphine withdrawal–induced negative affective states as well as the elevated *CRH* mRNA level. The precise mechanisms that allow CeA PV^+^ interneurons to modulate the activity of CRH^+^ neurons in the CeA during morphine withdrawal and how they ultimately regulate morphine withdrawal–induced affective-like behaviors need to be further investigated.

## Statement of interest

The authors declare no conflict of interest.

## Supplementary Material

Figure S1A–C
