# Supplementary figures and images for: Parvalbumin Interneurons of Central Amygdala Regulate the Negative Affective States and the Expression of Corticotrophin-Releasing Hormone During Morphine Withdrawal
Source: Int J Neuropsychopharmacol. 2016 Jul 6;19(11):pyw060. doi: 10.1093/ijnp/pyw060 (PMC5137277; doi:10.1093/ijnp/pyw060)

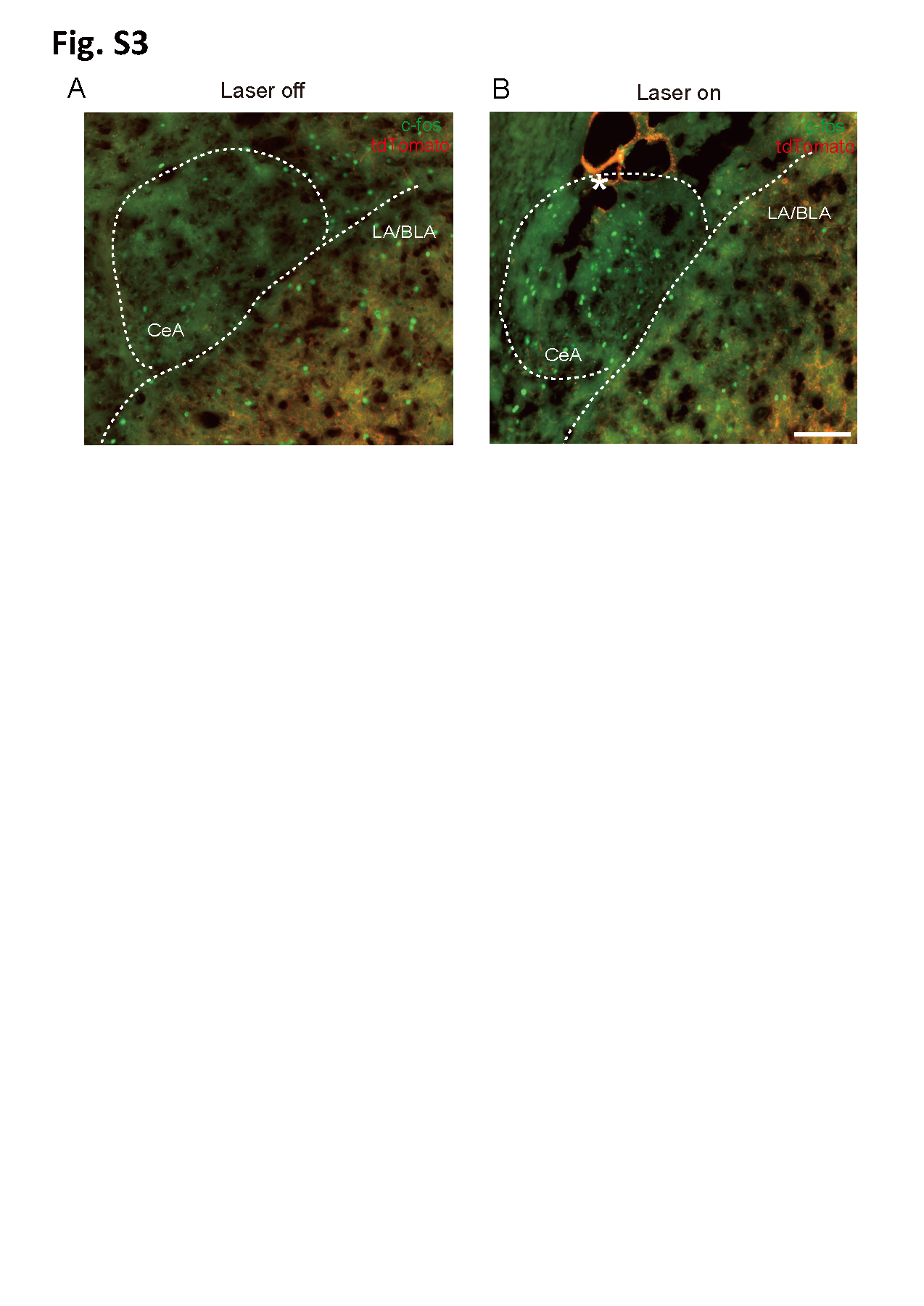

Supplement: Figure S1A–C [file 20160608_Fig_s3.tif]

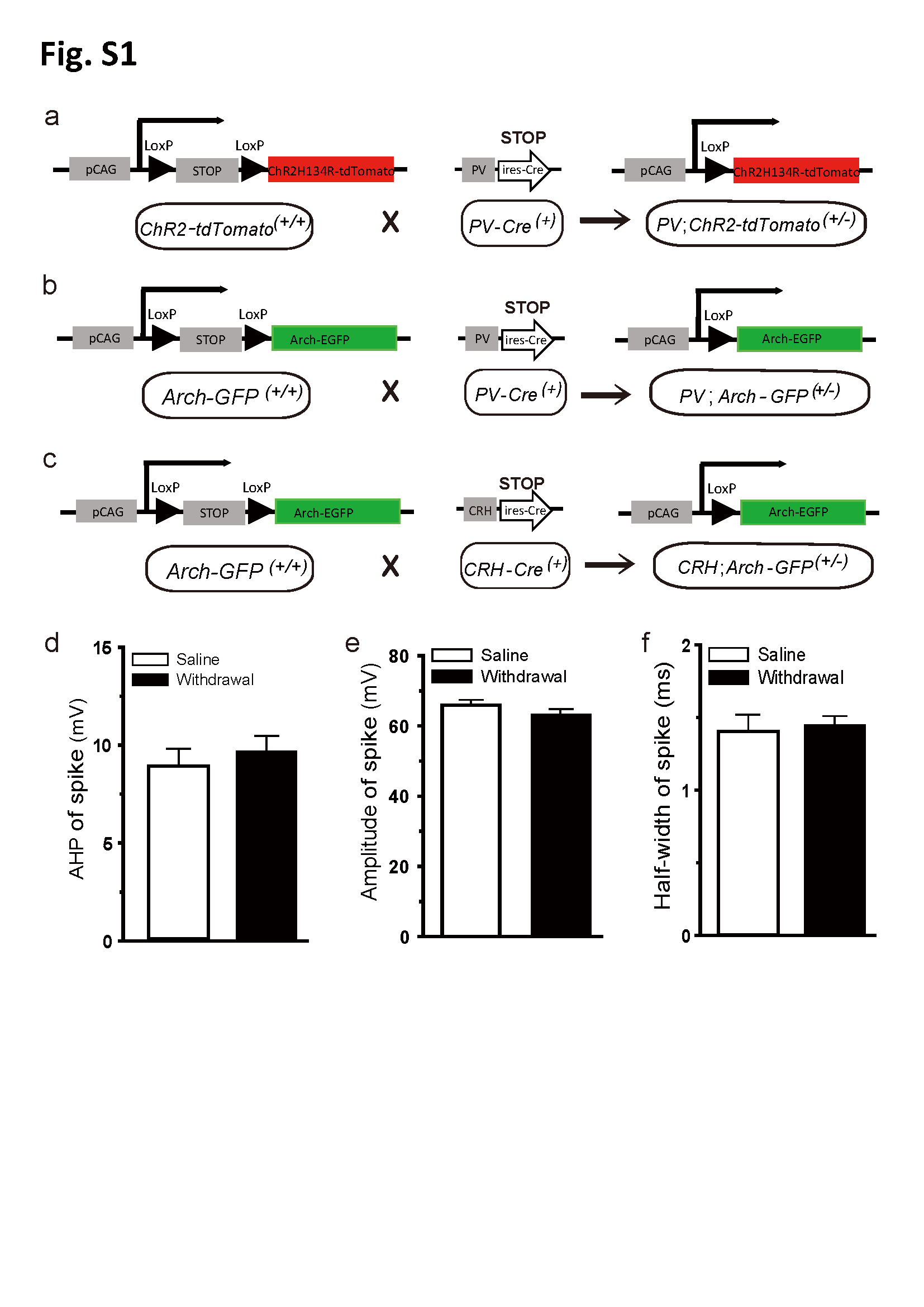

Supplement: Figure S1A–C [file 20160606_Fig_S1.tif]

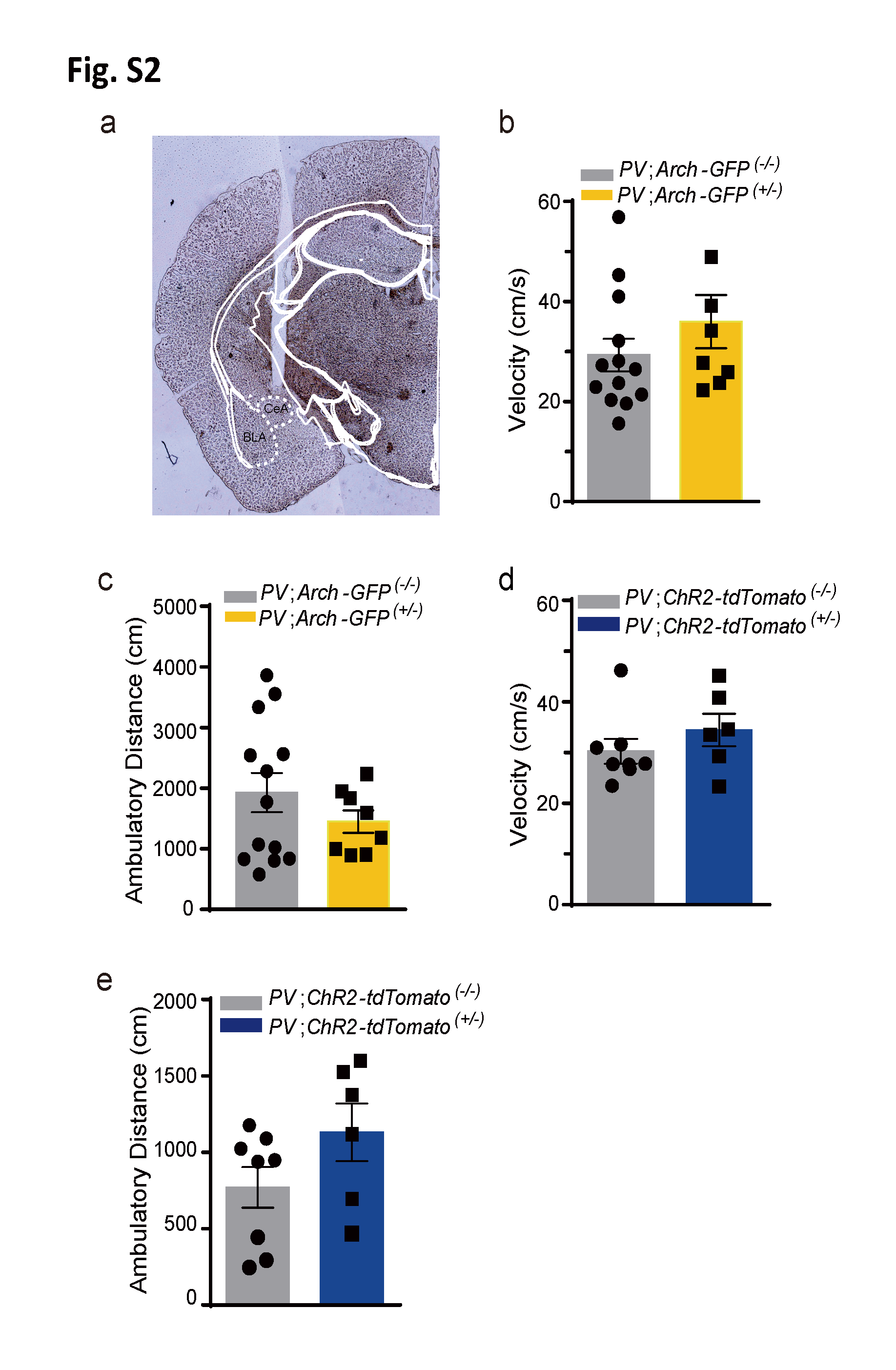

Supplement: Figure S1A–C [file 20160606_Fig_S2.tif]
