# Supplementary material for: Parvalbumin Interneurons of Central Amygdala Regulate the Negative Affective States and the Expression of Corticotrophin-Releasing Hormone During Morphine Withdrawal
Source: Int J Neuropsychopharmacol. 2016 Jul 6;19(11):pyw060. doi: 10.1093/ijnp/pyw060 (PMC5137277; doi:10.1093/ijnp/pyw060)
Supplement: Figure S1A–C [file Suppl_Fig_Legends.docx]

**Figure S1.** The breeding scheme of *PV; ChR2-tdTomato^(+/-)^*, *PV; Arch-GFP^(+/-)^* and *CRH; Arch-GFP^(+/-)^* mice, and the membrane parameters of PV^+^ neurons in CeA.

(a) The breeding scheme of *PV;ChR2-tdTomato^(+/-)^* mice. (b) The breeding scheme of *PV*;*Arch-GFP^(+/-)^* mice. (c) The breeding scheme of *CRH*; *Arch-GFP^(+/-)^* mice. (d-e) PV^+^ interneurons in the CeA showed no significant difference in the after-hyperpolarization potential (AHP) (d), the spike amplitudes (e), or the half-width of spike (f) of *PV;ChR2-tdTomato^(+/-)^* transgenic mice on the 6th day from the last saline or morphine exposure. Mann-Whitney U Test, *P< 0.05. Data are presented as mean ± s.e.m.

**Figure S2.** Optogenetic stimulation of CeA PV^+^ interneurons by the 594 nm or 473 nm laser alone does not influence the locomotion.

(a) The presentative histological image of the implantation of optical fibers into the CeA in the *PV*;*ChR2-tdTomato^(+/-)^* mice. (b-c) Bilaterally optogenetic inhibition of CeA PV^+^ interneurons by the 594 nm laser alone did not influence the travelling velocity and distance. (d-e) Bilaterally optogenetic activation of CeA PV^+^ interneurons by the 473 nm laser alone did not influence the travelling velocity and distance. Mann-Whitney U Test. Data are presented as mean ± s.e.m.

**Figure S3.** Optogenetic-activating the CeA PV^+^ interneurons increases the c-fos signaling of CeA in the *PV;ChR2-tdTomato^(+/-)^* mice.

(a-b) The c-fos signaling was dramatically increased in the CeA, but not in the BLA of the *PV;ChR2-tdTomato^(+/-)^* mice after the 30-min 473 nm laser stimulation in the CeA. Red: tdTomato, Green: c-fos, Scale bar: 100 µm. Arrows indicated the implantation trace of optical fiber inserted in the CeA.
